# Ectopic expression of *Jatropha curcas JcTAW1* improves the vegetative growth, yield, and drought resistance of tobacco

**DOI:** 10.1186/s12870-023-04085-2

**Published:** 2023-02-04

**Authors:** Qingyan Peng, Chang Liu, Zhurong Zou, Mengru Zhang

**Affiliations:** 1 First Affiliated Hospital of Kunming Medical University, Kunming, Yunnan China; 2 Engineering Research Center of Sustainable Development and Utilization of Biomass Energy, Ministry of Education, School of Life Sciences, Yunnan Normal University, Kunming, Yunnan China

**Keywords:** *JcTAW1*, Inflorescence architecture, Yield, Drought resistance, Transgenic tobacco

## Abstract

**Background:**

*Jatropha curcas* is a promising alternative bio-energy resource. However, underrun limited its broad application in the industry. Luckily, *TAW1* is a high-productivity promoting gene that increases the lateral branches by prolonging the identification of inflorescence meristems to generate more spikes and flowers.

**Results:**

In the current study, we introduced the Jatropha *JcTAW1* gene into tobacco to depict its functional profile. Ectopically expressed *JcTAW1* increased the lateral branches and ultimate yield of the transgenic tobacco plants. Moreover, the* JcTAW1* lines had significantly higher plant height, longer roots, and better drought resistance than those of wild-type (W.T.). We performed RNA sequencing and weighted gene co-expression network analysis to determine which biological processes were affected by *JcTAW1*. The results showed that biological processes such as carbon metabolism, cell wall biosynthesis, and ionization transport were extensively promoted by the ectopic expression of* JcTAW1*. Seven hub genes were identified. Therein, two up-regulated genes affect glucose metabolism and cell wall biosynthesis, five down-regulated genes are involved in DNA repair and negative regulation of TOR (target-of-rapamycin) signaling which was identified as a central regulator to promote cell proliferation and growth.

**Conclusions:**

Our study verified a new promising candidate for Jatropha productive breeding and discovered several new features of *JcTAW1*. Except for boosting flowering, *JcTAW1* was found to promote stem and root growth. Additionally, transcriptome analysis indicated that *JcTAW1* might promote glucose metabolism while suppressing the DNA repair system.

**Supplementary Information:**

The online version contains supplementary material available at 10.1186/s12870-023-04085-2.

## Introduction

*Jatropha curcas* is an attractive energy plant, due to its high-content biodiesel conversion of seeds, compatibility to crops without interference, and potential environmental protection [1]. However, low seed yield severely restricts its industry [2]. Introducing productive genes into Jatropha is a promising way for breeding high-yield varieties. Studies on plant flower development can provide high-yield candidate genes. FLOWERING LOCUS T/TERMINAL FLOWER 1 (FT/TFL1) gene family prevalently plays an essential regulatory role in flower induction by promoting or inhibiting the flowering transition in plants [3–7]. Li et al. studied the FT/TFL1 gene family in Jatropha and cloned its six members: *JcFT*, *JcTFL1a*, *JcTFL1b*, *JcTFL1c*, *JcMFT1*, and *JcMFT2* [8]. They found that Arabidopsis plants over-expressing *JcFT* showed extremely early flowering, while those of *JcTFL1b* or *JcTFL1c* showed prolonged vegetative growth and delayed flowering transition phenotype [8]. *AP1* (APETALA1) is also a key regulator in plant inflorescence architecture, which can control the specificity of both floral meristem and floral organ morphology [9–12]. It has been reported that *JcAP1* is only expressed in the inflorescence buds, female and male flowers of Jatropha [13]. Promoter activity analysis expressing *GUS*-fused *JcAP1* in Arabidopsis revealed that *JcAP1* is only active in inflorescence buds, stamens, and seeds [13]. Therefore, the *JcAP1* promoter can drive the specific expression of functional genes in Jatropha floral organs and seeds. Cytokinin (C.K.) regulates cell division and the activity of lateral meristem. Components of C.K. metabolic pathways such as isopentenyl transferase (IPT), cytochrome P450 monooxygenase CYP735A, and cytokinin oxidase/dehydrogenase (CKX) regulate its level [14]. A CRISPR-Cas9 based analysis found that Jccyp735a mutants showed severely retarded growth [15]. 6-BA(6-benzyl amino adenine) can promote bud formation and increase the number of buds. Chen et al. treated inflorescences of Jatropha with 6-BA, and screened out 34 genes related to flower development and 131 genes related to plant hormone metabolism [16]. Moreover, several studies constructed quantitative trait loci (QTL) mapping to provide valuable information to explore new high-yield candidate genes in Jatropha [17, 18].

Although there is a growing body of research on the increasing production of Jatropha in recent years, only some high-yield genes have been discovered. There is still a great need to find new candidate genes for the productive breeding of Jatropha. *TAWAWA1* (*TAW1*) was found to be a high-yield related gene that belongs to *Arabidopsis LSH1* and *Oryza G1* (ALOG) gene family [19]. The protein members of this family with a highly conserved ALOG domain have been found to play a vital role in regulating inflorescence development in land plants [20–23]. Like other ALOG members, *TAW1* was found to be a regulator that can suppress the adoption of floral fate in inflorescence and lateral meristems leading to production of more spikes and flowers [19]. Yoshida et al. found that the function-deficient *TAW1* mutants exhibited a reduced primary branching phenotype, while the gain-of-function mutant *taw1-D* showed more secondary and advanced branches. Further study revealed that in wild-type rice, *TAW1* gradually became undetectable during spikelet meristem initiation, while in the *taw1-D* mutant, it was still highly expressed in the same stage. *TAW1* has also been confirmed as a single over-dominant gene whose heterozygosity is effectual [24]. These results indicated that *TAW1* is a promising candidate gene for high-yield breeding. However, there are no studies of the *TAW1* gene in woody plants. To explore the function of the *TAW1* homolog genes in Jatropha, we cloned *JcTAW1* and introduced it into a widely used model plant, tobacco. To our surprise, except for the anticipated yield-improving effects, *JcTAW1* lines also exhibited significantly higher plant height, longer roots, and better drought tolerance than those of W.T. plants. To further study the underlying molecular mechanism, we performed an RNA sequencing and weighted gene co-expression network analysis (WGCNA) [25].

## Materials and methods

### Plant materials and growth conditions

Jatropha seeds were surface-sterilized with 1% CuSO_4_ and placed on six layers of wet filter papers to germinate in dark for five days. Then the sprouted seeds were planted in matrix soil mixed with perlite, vermiculite and humous at the ratio of 1:1:1. The soil-grown *J. curcas* was cultivated in a growth chamber at 26 ± 1 °C under a light/dark regime of 16/8 h. 0.1 mg leaf pieces were clipped from Jatropha seedlings with 2–4 emerging true leaves and used to extract genomic DNA as previously described [26].

This study used the K326 tobacco cultivar. Tobacco culturing was maintained in a growth chamber at 25 ± 1 °C under a light/dark regime of 16/8 h.

### Gene amplification and vector construction

*JcTAW1* gene sequence was retrieved with the query of rice TAW1 protein from the whole-genome shotgun contigs (WGS) of Jatropha via the tblastn tool of NCBI. Similar to the rice *TAW1* gene, *JcTAW1* has no introns, so it can be amplified directly from Jatropha genomic DNA extracted as previously described [26]. *JcTAW1* gene was amplified by two rounds of nested PCR using Phusion high-fidelity DNA polymerase (NEB). The first round of PCR used the flanking gene primers JcTAW-Fw, JcTAW-Rv and Jatropha genomic DNA as the template. The crude product was 100-fold diluted as a template for the second round of PCR with the gene-specific primer pair JcTAW1-5Kn/JcTAW1-3Sc. Then the purified amplicon fragment was cloned into the plant binary vector pBI121 (Clontech) by *KpnI* and *Sac*I (NEB) digestions to generate plant expression vector *pBI121 (JcTWA1)*. All primers used for vector construction are listed in Table S1.

### JcTAW1 bioinformatic analysis

Based on the nucleotide sequence of the *JcTAW1* gene, we predicted its nuclear localization signal (NLS) sequence using the online server http://www.moseslab.csb.utoronto.ca/NLStradamus [27]. With the deduced protein sequence of *JcTAW1*, we constructed its phylogenetic tree using Geneious R10.

### Generation of transgenic tobacco plants

*Agrobacterium tumefaciens* strain LBA4404 carrying plant expression vector *pBI121 (JcTAW1)* was transformed into sterile tobacco explants by leaf disc infiltration. The transformed explants were selected on kanamycin-containing M.S. solid media. Positive transgenic tobacco lines were strictly identified from kanamycin-resistant transformants by multiplex genomic PCR with primer pairs JcTAW1-5Kn/JcTAW1-3Sc, 35Spro-Fw/JcTAW1-3Sc, and JcTAW1-5Kn/NosTer-Rv. JcTAW1-5Kn and JcTAW1-3Sc are gene-specific primers, 35Spro-Fw and NosTer-Rv are promoter or terminator primers. The expression pattern of *JcTAW1* gene in transgenic tobacco plants was profiled by RT-PCR with primers JcTAW1-iFw, JcTAW1-iRv. The results were normalized to 18 s rRNA. Primers used for transgenic plant identification and RT-PCR are listed in Table S1.

### Phenotypic analysis of transgenic tobacco

The phenotype of W.T. and three independent *JcTAW1* transgenic lines of T1 generation were profiled, including bud number, lateral branch number, number of buds on each branch, plant height, inflorescence morphology, number of seeds per bud, thousand kernel weight, yield per plant, primary root length, and number of lateral roots. Three repetitions were used for all observed parameters.

#### Plant height measurement and inflorescence morphology profile

The height of each transgenic and W.T. plant was measured after the floral transition since then tobacco stem length will not change much. Photos of the mature status and the inflorescence morphology of transgenic and W.T. plants at the same stage were compared.

#### Measurement of buds, lateral branches, and seeds per capsule

The number of buds and lateral branches of transgenic and W.T. plants were recorded at the end of inflorescence development. The average number of buds on each lateral branch was calculated. Seeds of each capsule were collected before splitting, and the thousand kernel weight was calculated.

#### Measurement of primary roots and number of lateral roots

Roots of transgenic and W.T. mature plants at the same stage were isolated from the soil and washed carefully. The length of primary roots and the number of lateral roots were measured.

### RNA sequencing

The total RNA of flowers and roots of W.T. and three independent *JcTAW1* transgenic lines were extracted using RNAiso Plus (TAKARA), used to construct cDNA libraries, and then sequenced on the Illumina HiSeq2500 platform. Clean data were obtained by removing adapter sequences and low-quality raw data.

### Differential gene expression (DGE)

The differentially expressed genes (DEGs) of flowers and roots between *JcTAW1* line and W.T. plant were identified from the gene expression data using the DESeq R package (version: 1.38.0) [28]. Genes with |log_2_ fold change (F.C.)|> 1 and an FDR < 0.05 were identified as DEGs.

### WGCNA

The RNA-seq clean data of flowers and roots from three independent *JcTAW1* lines and two independent W.T. plants were used for WGCNA with R package ‘WGCNA’ [25]. To construct the co-expression network of flower and root, respectively, we first constructed a hierarchical clustering tree through an expression similarity matrix. We calculated the scale-free topology fit index as a soft-thresholding power and transformed the similarity matrix into an adjacency matrix. After calculating intramodular connectivity between genes, the adjacency matrix was transformed into a topological overlap matrix (TOM). Then genes with similar expression profiles were divided into different modules (with a minimum of 30 genes per module). Through the dynamic tree cut method, TOM-based dissimilarity (dissTOM) was used to construct the hierarchical clustering tree and calculate the module eigengene (M.E.). M.E. is defined as the first principal component of a specific module and represents the overall gene expression level of the module.

### Identification of significant phenotypic modules

The correlations between the modules and phenotypic traits were used to estimate the module-trait associations. We selected a flower key module and a root key module respectively, using the Pearson correlation coefficients between the M.E.s of each module and each phenotypic trait (*P* < 0.05 was set as the significance threshold).

### Functional enrichment analysis

The cluster Profiler R package (version: 3.16.0) was used to conduct gene ontology (G.O.) annotation and Kyoto encyclopedia of gene enrichment analysis of all genes in the two key modules [29]. *P* < 0.05 was set as the significance threshold.

### Identification of hub genes

The intramodular connectivity of genes in the brown module of the root and yellow module of the flower were calculated using module eigengene-based connectivity (kME). Hub genes in the key modules were screened with a threshold of kME > 0.9.

### Drought assays

Thirty-day-old soil-grown W.T. tobacco plants and three independent *JcTAW1* transgenic lines were subjected to severe drought stress by withholding water for 21 days, followed by seven days of re-watering. Photos were taken, and the root length was measured.

### Statistical analysis

Data were analyzed using GraphPad Prism 5 software (GraphPad Software Inc.) and represented as mean ± S.D. Analyses involving three or more group data sets were performed with repeated measures of one-way ANOVA with Tukey's multiple comparison test. Significance was set as a *p*-value of less than 0.05.

## Results

### Expression of *JcTAW1* increases the yield of the transgenic tobacco lines

The *JcTAW1* gene sequence was obtained by the tblastn tool of NCBI against rice TAW1 protein. The coding sequence (CDS) of the *JcTAW1* gene was 642 bp, encoding a 213 aa protein termed JcTAW1 (Figure S1). JcTAW1 is a transcript factor, so we predicted its nuclear localization signal (NLS) (Figure S1). Furthermore, a phylogenetic tree was used to analyze the family of the* JcTAW1* gene, which indicates it belongs to the ALOG family. Moreover, its orthologous genes exist in both monocotyledons and dicotyledons. JcTAW1 is evolutionarily closest to TAW1 in *Euphorbiaceae* plants (such as *Ricinus communis*) and *Solanum* plants (Figure S2). Since there are at least 10 to 11 ALOG genes in rice and Arabidopsis genomes, an almost equal number of ALOG members might be found in Jatropha genome annotated recently. So we further retrieved in Jatropha and rice specific protein databases with rice OsTAW1 and Jatropha JcTAW1 as query sequences, alternately using the tool of blastp. There are 8 ALOG members in the Jatropha genome. To our surprise, the ‘TAW1’ homologue in Jatropha closest to rice OsTAW1 is not the ‘JcTAW1’ characterized in this study, but LSH1 | XP_012089329 (probably because the annotation of Jatropha genome was not finished when we started this work), and in rice, the closest homologous ‘TAW1’ to ‘JcTAW1’ is G1-like 6 | XP_015624971 (Figure S3).

**Fig. 1:**
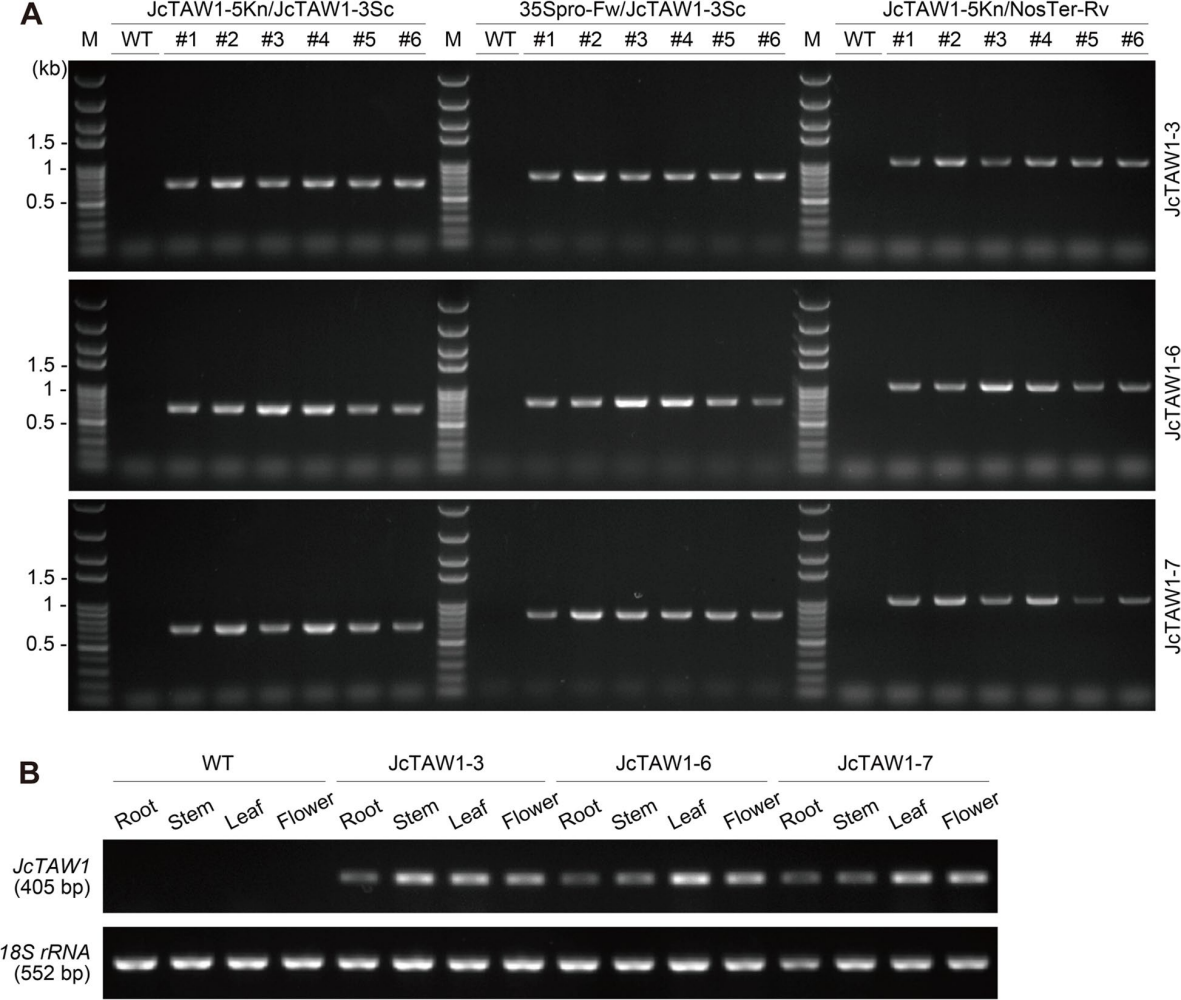
*JcTAW1* expression profile in verified transgenic lines. **A** PCR identification of *JcTAW1* transgenic tobacco lines with three pairs of primers. **B** The mRNA expression pattern of transgenic lines JcTAW1-3/6/7 analyzed by RT-PCR. The original gel graphs are presented in Figure S7

The T1 *JcTAW1* transgenic tobacco lines were strictly identified with three pairs of primers (Fig. 1A), and the *JcTAW1* expression pattern in transgenic lines was further determined by RT-PCR (Fig. 1B). *JcTAW1* mRNA was detected in roots, stems, leaves, and flowers of three transgenic tobacco lines (JcTAW1-3/6/7), and the expression levels in leaves and flowers were generally higher than those in roots and stems (Fig. 1B). In contrast, there were no *JcTAW1* mRNAs detected in any tested tissues of W.T. plants.

**Fig. 2:**
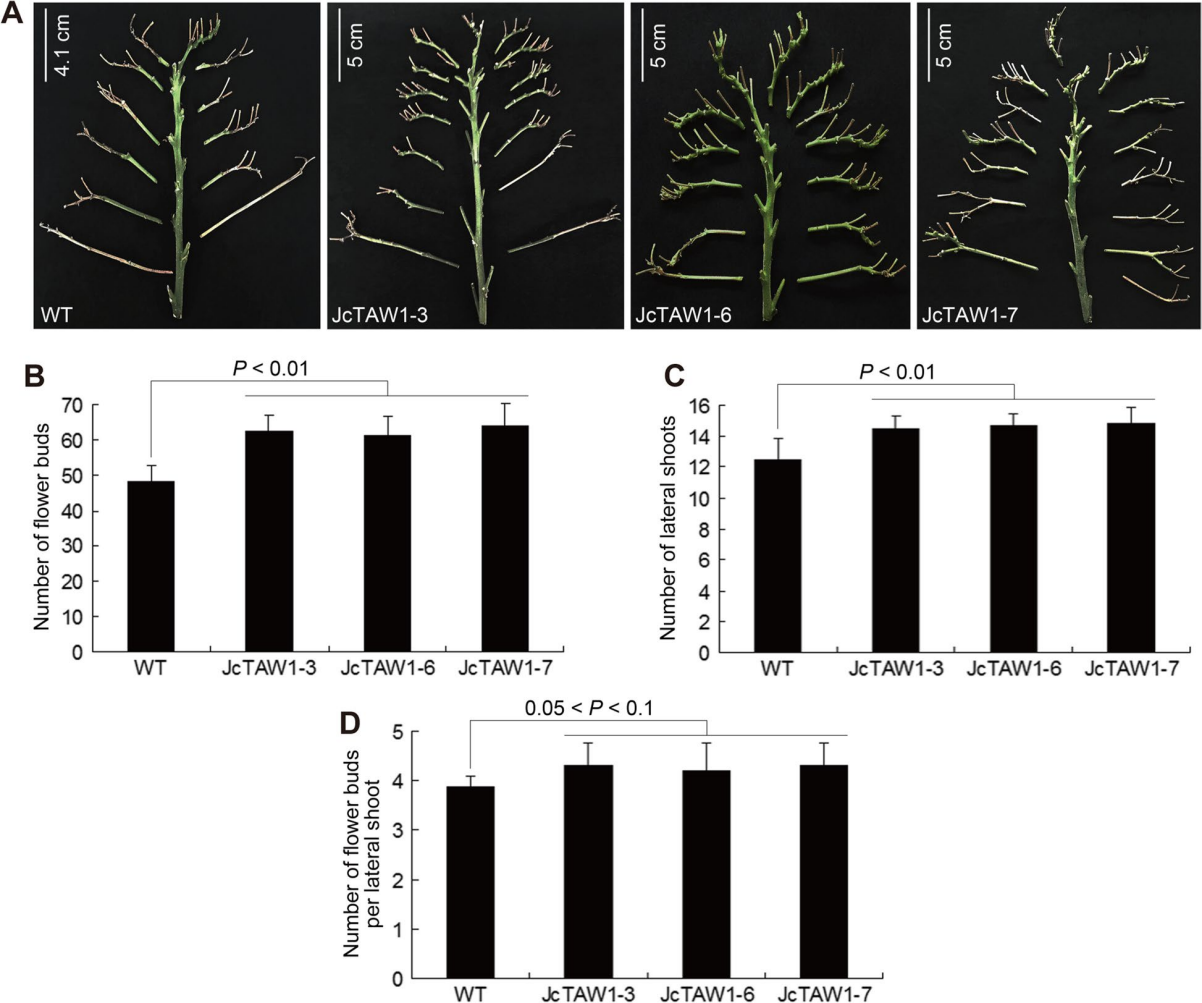
Ectopically expressed *JcTAW1* enhances the inflorescence architecture and increases the yield of the transgenic tobacco lines. **A** Inflorescence comparison of W.T. and transgenic plants. **B** Measurement of the number of flower buds. **C** Measurement of the number of lateral shoots. **D** Measurement of the number of flower buds per lateral shoot. Data are represented as mean ± S.D

The canonical function of the *TAW1* gene is to suppress the transition to branch meristem identity, which promotes the emergence of more flowers. We reasoned that *JcTAW1* transgenic tobacco plants might exhibit an increased flowering phenotype. So we compared the inflorescences of transgenic and W.T. plants. In deed, plants of all three different transgenic lines harbored more flowers than W.T. tobaccos (Figure S4). A dissection analysis displayed that, the lateral branch number of transgenic lines was comparable to that of W.T. tobacco, but the secondary branches of transgenic plants were significantly more (Fig. 2A and C). W.T. plants usually generate 1 to 4 buds on each lateral branch, whilst  *JcTAW1* transgenic plants can produce 3 to 6 buds. Moreover, the transgenic line JcTAW-7 even reiterated the pattern of primary branch on the lateral branch (Fig. 2A and C). As a result, despite with almost equal number of lateral branch, the buds on each lateral branch of transgenic lines were evidently more than that of W.T. The total number of buds per transgenic tobacco plant were 1.32 times as more as that of W.T. plants (Fig. 2B and D).

**Fig. 3:**
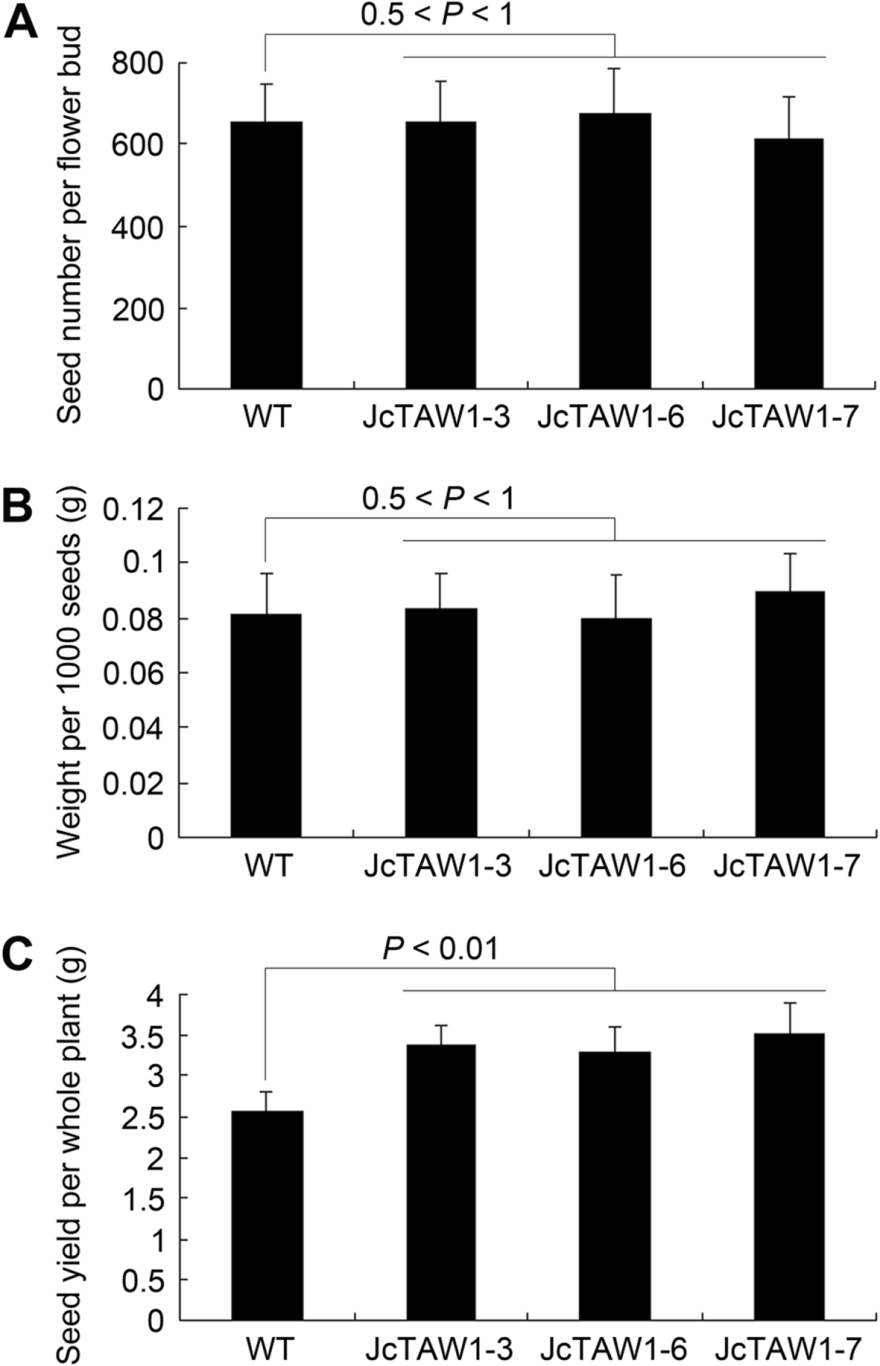
Ectopically expressed *JcTAW1* increases the yield of transgenic tobacco lines. **A** Measurement of the seed number per flower bud. **B** Measurement of the weight per 1000 seeds. **C** Measurement of seed yield (g/plant). Data are represented as mean ± S.D

We collected the mature capsules of *JcTAW1* transgenic lines and W.T. plants before splitting to record the number of seeds in each capsule, and found out that the seeds of transgenic lines were 1.31 times as more as that of W.T. plants (Fig. 3C). However, there was no significant difference in seed number per flower bud and weight per 1000 seeds (Fig. 3A and B). These results demonstrate that the ectopic expression of *JcTAW1* can increase the yield of transgenic tobacco plants by promoting the production of more flowers.

### Expression of *JcTAW1* enhances the vegetative growth and root architecture of the transgenic tobacco lines

**Fig. 4:**
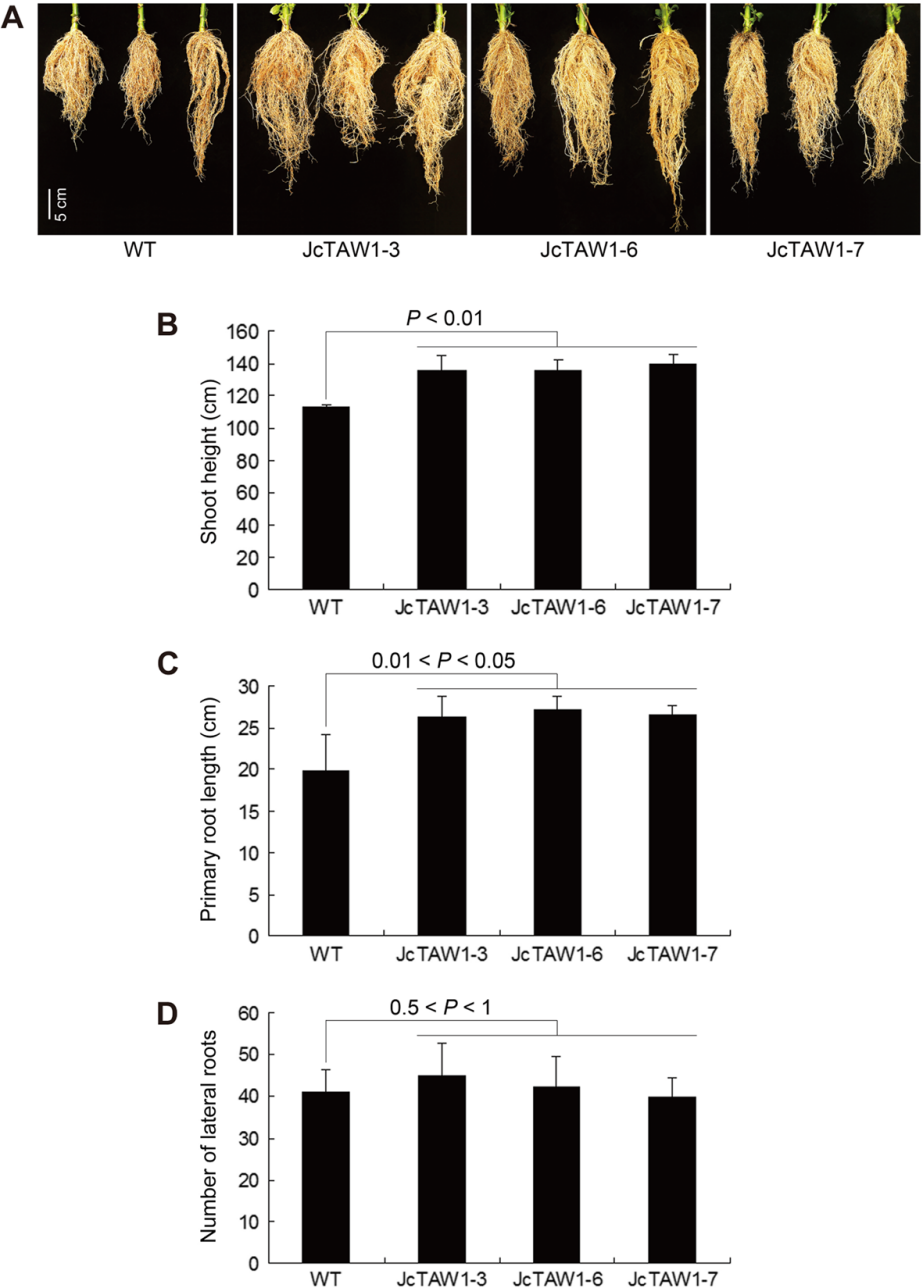
Ectopic *JcTAW1* enhances the vegetative growth and root development of transgenic tobacco lines. **A** Comparison of the root morphology of W.T. and transgenic plants. **B** Measurement of the plant  height. **C** Measurement of the primary root length. **D** Measurement of the lateral root number. Data are represented as mean ± S.D

We noticed that in addition to increased number of buds, the T1 *JcTAW1* transgenic tobacco lines had significantly longer stem than W.T. plants (Figs. 4B and S5). The average height of transgenic plants was 1.22 times as long as that of W.T. plants, suggesting that the ectopic expression of *JcTAW1* can promote the vegetative growth in  tobacco. The higher plant height of *JcTAW1* lines may be due to a better-developed root system and thus better uptake of nutrients. Probably,  *JcTAW1* has a similar effect on roots as on flowers that prolongs the transition of root meristem and generates more secondary, tertiary, or inferior roots. To verify our conjecture, we compared the root morphology. As shown in Fig. 4A, all transgenic lines  (JcTAW1-3/6/7) had more extensive root system than W.T. plants. The average length of primary roots of *JcTAW1* transgenic plants was significantly longer (1.35 times) than that of W.T. tobaccos (Fig. 4A and C). However, to our surprise, the lateral roots of transgenic plants were only 1.09 times as long as that of W.T. (Fig. 4D). The results suggested that JcTAW1 might only promote root and stem cell proliferation. Whether JcTAW1 can regulate the root architecture by means  in flower needs further studies.

### Introduction of *JcTAW1* alters the gene expression profile of the transgenic tobacco lines

Interestingly, the ectopically expressed *JcTAW1* affects the inflorescence architecture and the development of the entire transgenic plants, especially the root system. To find out which bio-processes of the transgenic plants had been altered by the *JcTAW1* gene, we compared the mRNA expression profiles between the transgenic and W.T. plants through an RNA sequencing analysis.

#### Identification of DEGs

**Fig. 5:**
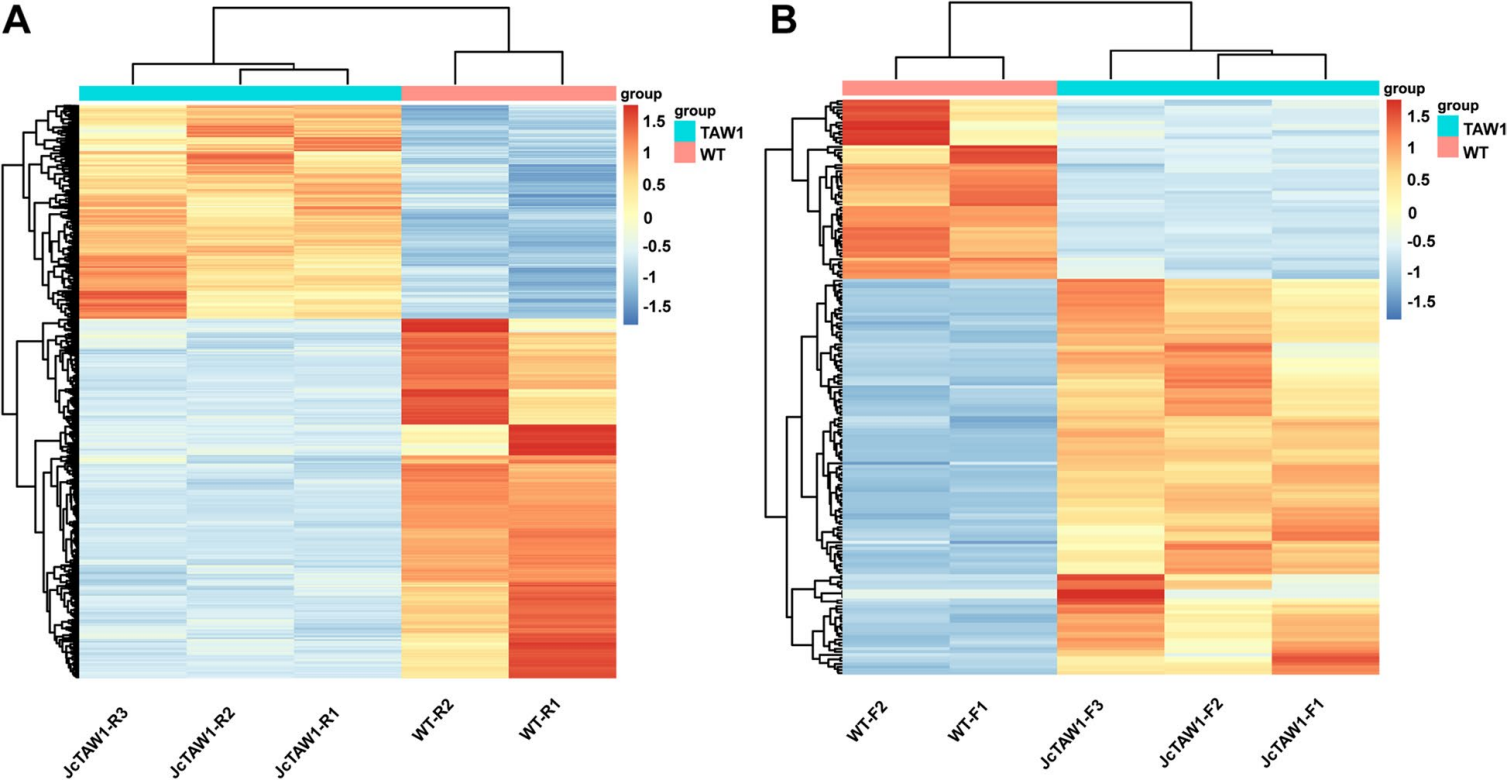
Analysis of DEGs in root and flower tissues between three independent *JcTAW1* transgenic lines and W.T. plants. **A, B** Heatmap of the differentially expressed mRNAs (FDR < 0.05; |Log_2_ F.C.|> 1) in roots and flowers, respectively

Since the expression patterns in the root and flower were vastly different, we identified the DEGs of the root and flower, respectively. The RNA-seq data of the roots and flowers from three T1 transgenic lines (JcTAW1-3/6/7) and two independent W.T. plants were used to find DEGs. A total of 4617 (2892 up-regulated and 1725 down-regulated) DEGs in root and 189 (59 up-regulated and 130 down-regulated) DEGs in flower were screened out at the threshold of |log2 F.C.|> 1 and FDR < 0.05, and selected for subsequent analysis (Fig. 5A and B). From the Heatmap of the DEGs in flower and root tissues, it is clear that the transgenic and W.T. pants belong to distinct groups (Fig. 5A and B), and the repeatability of samples in each group is very high.

#### Identification of significant phenotypic modules

**Fig. 6:**
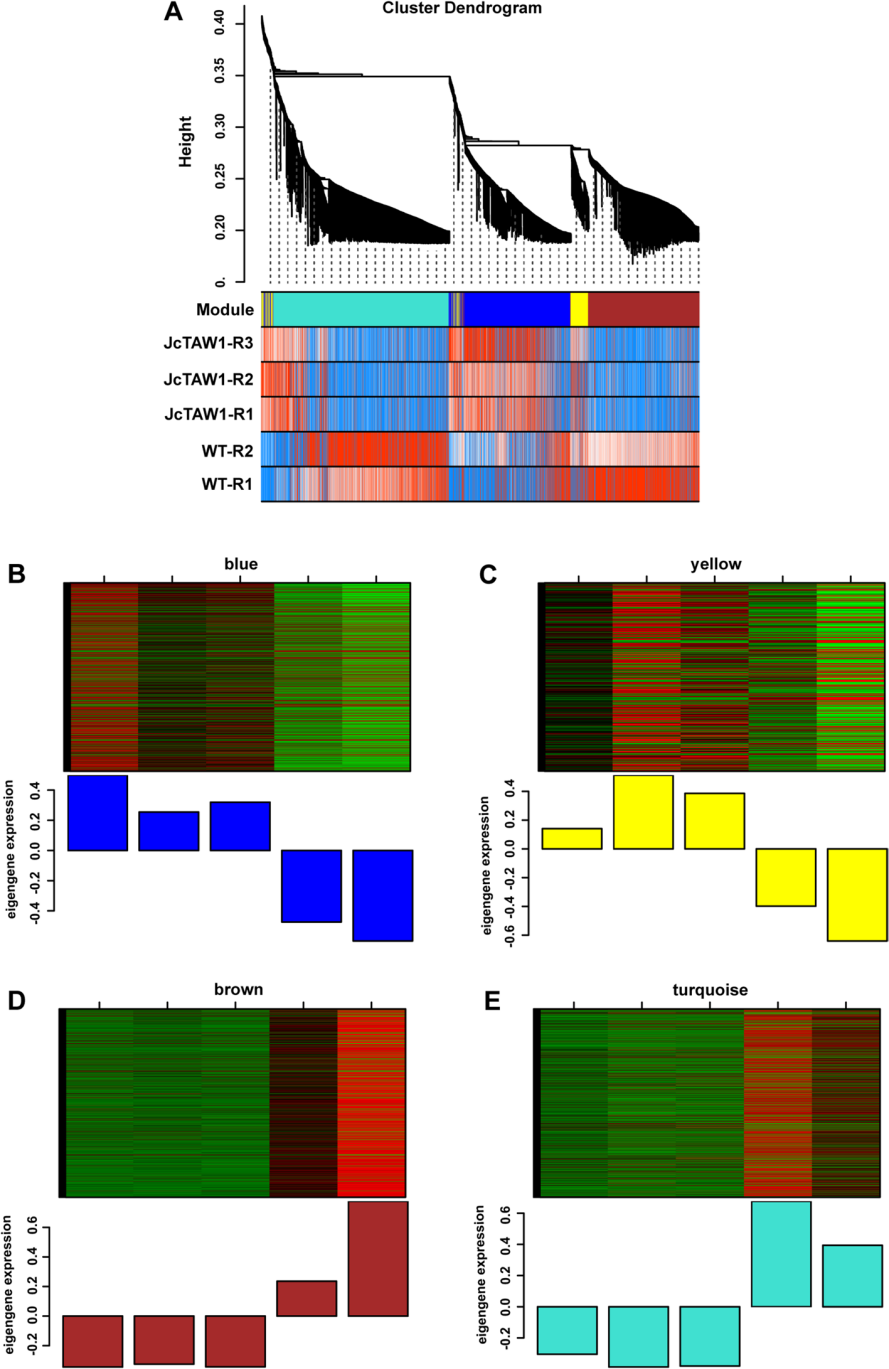
Construction of weighted co-expression network and module detection of the root. **A** Clustering dendrograms of DEGs in root tissues. Each color represents one specific co-expression module, and the branches above represent genes. Blue (**B**), yellow (**C**), brown (**D**), and turquoise (**E**) modules were identified and the expression levels of genes in modules versus each sample and M.E. values of modules versus each sample were calculated

**Fig. 7:**
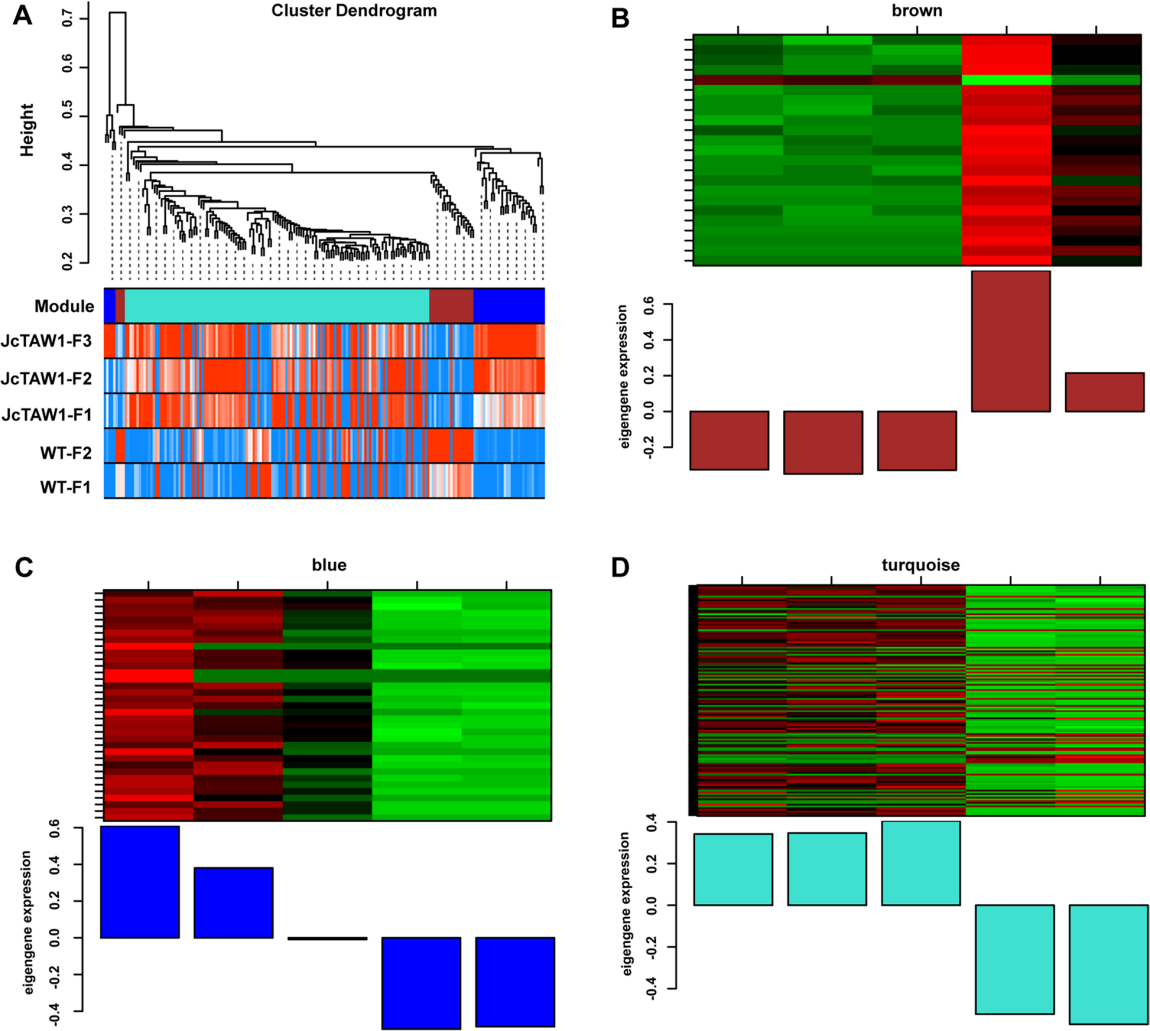
Construction of weighted co-expression network and module detection of the flower. **A** Clustering dendrograms of DEGs in flower tissues. Each color represents one specific co-expression module, and the branches above represent genes. Brown (**B**), blue (**C**), and turquoise (**D**) modules were identified and the expression levels of genes in modules versus each sample and M.E. values of modules versus each sample were calculated

**Fig. 8:**
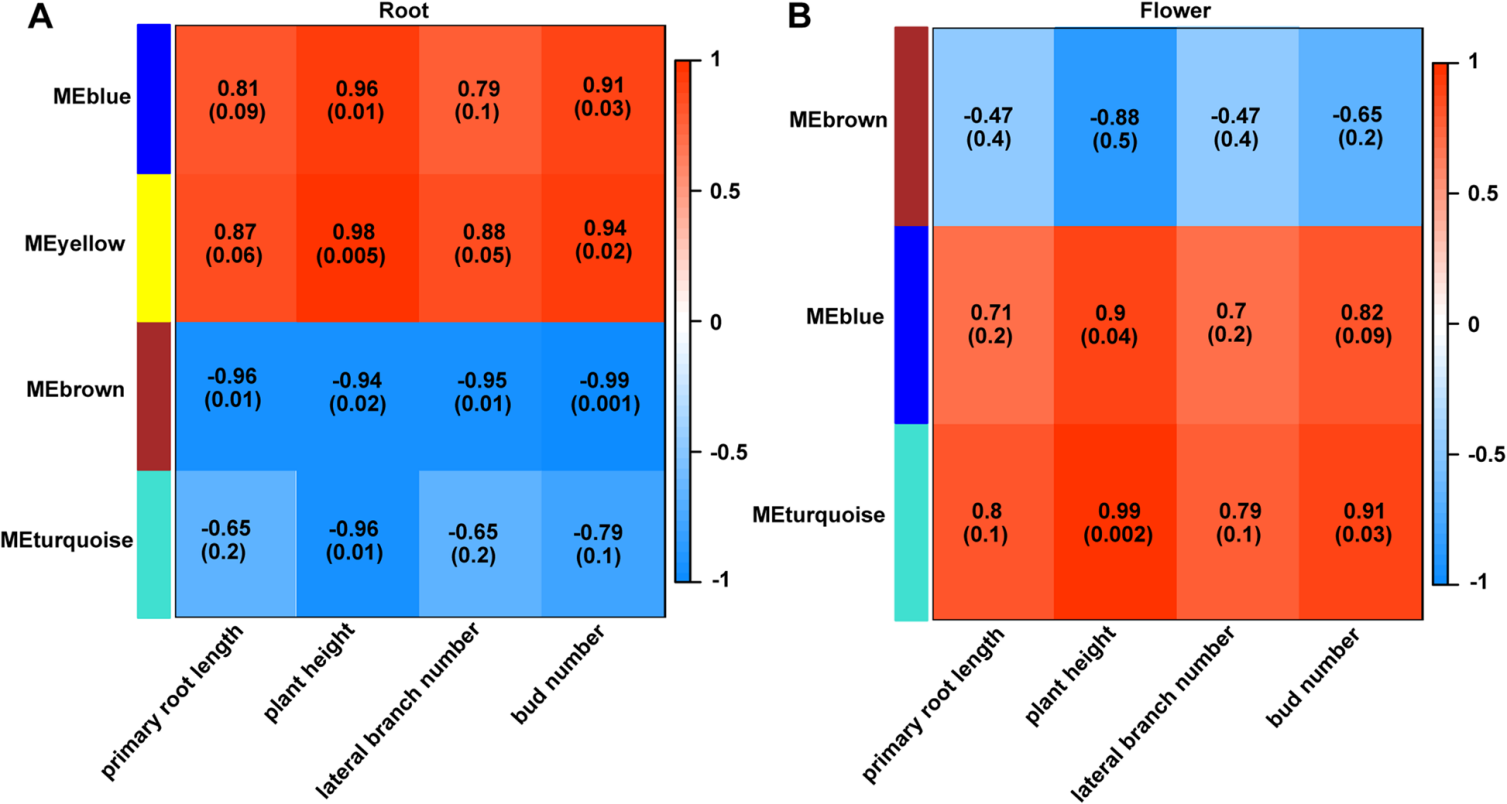
The relationships between modules and phenotypic traits in the root (**A**) and flower (**B**). Each row represents a module eigengene, each column represents a phenotypic trait, and each cell consists of the corresponding correlation and *p*-value. Red represents a positive correlation, and blue represents a negative correlation

According to the rule of a scale-free network, the soft-thresholding power, β = 5 (scale-free *R*^2^ = 0.99) and β = 7 (scale-free *R*^2^ = 0.85) were chosen respectively to construct root and flower gene co-expression networks. After determining the soft-thresholding power, β, the co-expression networks of 1725 root DEGs and 189 flower DEGs were constructed and hierarchical clustering dissTOM trees of root and flower DEGs were obtained, respectively (Figs. 6A and 7A). Four modules of root DEGs and three modules of flower DEGs were identified, and M.E.s of all modules were calculated (Fig. 6B, C, D, E, and Fig. 7B, C, D). The key modules closely related to the main phenotypic traits have been selected by calculating the Pearson correlation coefficients for the M.E.s of all modules and the phenotypic information. In terms of the root, the blue module was significantly associated with the plant height (*r* = 0.96, *P* = 0.01) and the bud number (*r* = 0.91, *P* = 0.03); the yellow module was significantly associated with the plant height (*r* = 0.98, *P* = 0.005), the lateral branch number (*r* = 0.88, *P* = 0.05) and the bud number (*r* = 0.94, *P* = 0.02); the brown module was significantly associated with the primary root length (*r* = -0.96, *P* = 0.01), the plant height (*r* = -0.94, *P* = 0.02), the lateral branch number (*r* = -0.95, *P* = 0.01) and the bud number (*r* = -0.99, *P* = 0.001); the turquoise module was significantly associated with the plant height (*r* = -0.96, *P* = 0.01). In conclusion, only the brown module was significantly associated with all four phenotypic traits, so we chose the brown module as the key module of the root DEGs (Fig. 8A). For the flower, the brown module was significantly associated with the plant height (*r* = -0.88, *P* = 0.05); the blue module was significantly associated with the plant height (*r* = 0.9, *P* = 0.04); the turquoise module was significantly associated with the plant height (*r* = 0.99, *P* = 0.002) and the bud number (*r* = 0.91, *P* = 0.03); so the turquoise module was chosen as the key module of the flower DEGs (Fig. 8B).

#### The effect of *JcTAW1* on the biological processes of transgenic tobacco lines

**Fig. 9:**
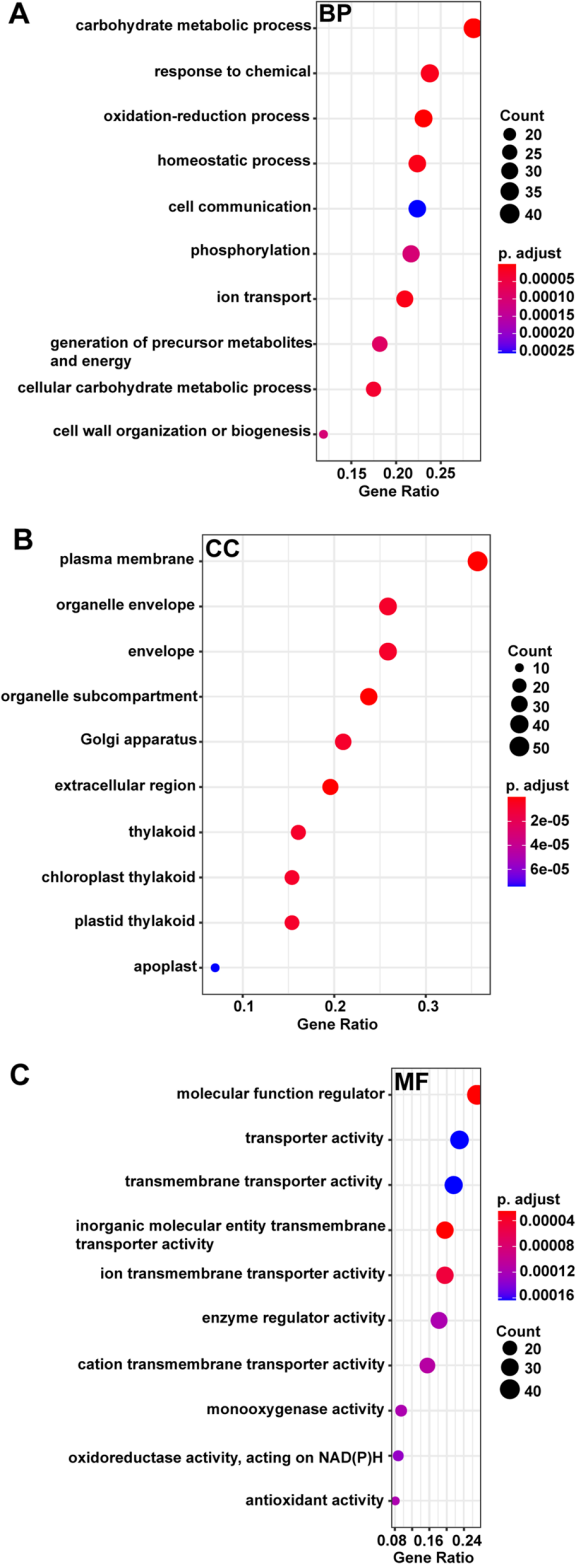
Gene ontology (G.O.) enrichment analysis of the top ten enriched biological processes (B.P.) (**A**), the top ten enriched cellular components (C.C.) (**B**), and the top ten enriched molecular function (M.F.) (**C**)

Highly co-expressed genes in the key module are more likely to be of biological significance. To discove which bio-processes of transgenic tobacco plants were affected by the ectopic expression of *JcTAW1*, we performed G.O. (biological process, cellular component, and molecular function) enrichment analysis of genes in the two phenotypic significant modules altogether. In the G.O. functional enrichment analysis, the top 10 enriched biological processes were ‘carbohydrate metabolic process’, ‘oxidation–reduction process’, ‘homeostatic process’, ‘ion transport’, ‘response to chemical’, ‘cellular carbohydrate metabolic process’, ‘generation of precursor metabolites and energy’, ‘phosphorylation’, ‘cell wall organization or bio-genesis’, and ‘cell communication’ (Fig. 9A and Table S2). Besides, genes in the key module are mainly located on biomembranes or extracellular region (Fig. 9B and Table S3) and act as molecular function regulator, inorganic molecular transmembrane transporter, oxidoreductase, and roles like these. The top ten enriched molecular functions of the co-expressed genes were coordinated with that of the biological processes (Fig. 9C and Table S4).

**Fig. 10:**
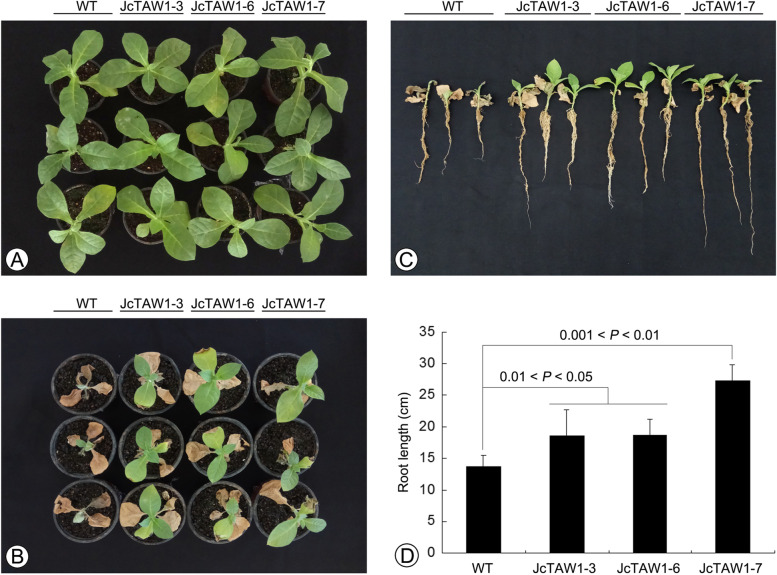
Ectopic expression of *JcTAW1* improves the drought resistance of transgenic tobacco lines. **A** The transgenic lines JcTAW1-3/6/7 and W.T. plants before drought assay. **B** The transgenic lines JcTAW1-3/6/7 and W.T. plants after 21-day withholding water and 7-day re-watering. **C** and **D** Comparing the root length of the transgenic lines JcTAW1-3/6/7 and W.T. plants in drought assay. Data are represented as mean ± S.D

Seven hub genes met kM.E. > 0.9 in the key modules were screened. Up-regulated genes are *XTH* and *PR0*; down-regulated genes are *LOC107823473*, *Ntubc2*, *LOC107786522*, *LOC107761646*, and *LOC107787142*. The two up-regulated genes are involved in the carbohydrate metabolic process and cell wall bio-genesis, the five down-regulated genes are involved in DNA repair and the negative regulation of TOR signaling.

### Expression of *JcTAW1* improves the drought resistance of the transgenic tobacco lines

As described above, the root system of transgenic lines was developed much better than that of W.T. plants, so we supposed that the *JcTAW1* plants might have a better drought resistance than W.T. To verify our point, a drought assay was carried out that 30-day-old W.T. and transgenic plants of three independent lines were subjected to severe drought stress. The results were in line with our prediction that after withholding water for 21 days and 7-day re-watering, W.T. plants could not recover from wilting, while all three independent transgenic lines contrastively showed higher survival rate and larger biomass (Fig. 10A and B). To verify the relationship between root architecture and drought resistance, we further measured the root length of the transgenic and W.T. plants under the drought assay. We found that the average root length of three transgenic lines JcTAW1-3/6/7 was 1.36, 1.35, and 1.86 times respectively, as long as that of W.T. plants (Fig. 10C and D). The results demonstrated that the *JcTAW1* gene could promote the root development of the transgenic tobacco lines, contributing to an enhanced drought resistance.

## Discussion

Jatropha is an organic oil crop that has been recognized as a valuable new energy source in recent years. However, the low yield of Jatropha limited its industrialization. Generating new varieties of Jatropha with increased yield is a solution. *TAW1* is a single over-dominant gene that can promote high-yield as a heterozygote, so it is a promising candidate for productive Jatropha breeding. In the current study, the *TAW1* gene of the woody angiosperm Jatropha, *JcTAW1*, was cloned for the first time, and the morphological characteristics of *JcTAW1* transgenic tobacco lines were analyzed. Consistent with previous studies, *JcTAW1* transgenic lines harbor more buds than W.T. plants, and thus more seeds. Our study confirmed that the Jatropha *JcTAW1* gene has a dominant yield-increase function. Besides, there were also some unexpected discoveries that the transgenic tobacco lines had a growth advantage over W.T. plants, with taller plant heights. The growth advantage might be due to a more developed root system. *JcTAW1* may prolong the root meristem’s activity and promote secondary lateral root generation. Nevertheless, *JcTAW1* did not increase the lateral root number, but only strongly increased the primary root length.

The different effects of *JcTAW1* on flowers and roots imply it has a complex function in regulating plant development. So we performed an RNA-seq analysis on flower and root tissues between *JcTAW1* transgenic lines and W.T. plants to explore the underlying mechanism. All three independent *JcTAW1* transgenic tobacco lines were highly consistent. They were recognized belonging to groups distinguished from W.T. plants both in root and flower tissues through the clustering analysis, indicating that the ectopic *JcTAW1* substantially impacts the gene expression patterns of transgenic tobacco plants. Although WGCNA is supposed to be more suitable for analyzing multi-sample data of more than 15 samples in general, we believed that our ‘small-sample’ is also applicable, based on the following two points: (1) the transgenic and W.T. plants belonged to distinct groups by clustering analysis; (2) all three *JcTAW1* transgenic lines and two independent W.T. plants were highly repetitive within each group. These observations suggested that the gene expression pattern altered by *JcTAW1* would conform to the scale-free network to a large extent, and the correlation networks impacted by ectopically expressed *JcTAW1* could be analyzed by WGCNA. The scale-free topology fit index calculation later confirmed that the soft-thresholding power of root and flower gene co-expression networks were β = 5 (scale-free *R*^2^ = 0.99) and β = 7 (scale-free *R*^2^ = 0.85), respectively. Highly correlated genes were identified in the same modules. Furthermore, significant phenotypic modules of the root and flower were determined by calculating the Pearson correlation coefficients for the M.E.s of all modules and the main phenotypic traits we were interested in (i.e. primary root length, plant height, lateral branch number and bud number). The Brown module of the root exhibited the highest levels of correlation with all of the phenotypic traits (except plant height), and turquoise module of flower exhibited the highest levels of correlation with plant height and bud number, so these two modules were chosen as the phenotypic significant modules of root and flower, respectively.

We performed function enrichment analysis within the two key modules to explore which bio-processes associated with the phenotypic traits were influenced by JcTAW1. It is worth noting that among the top ten enriched biological processes, three items ‘carbohydrate metabolic process’, ‘cellular carbohydrate metabolic process’ and ‘generation of precursor metabolites and energy’ were involved in energy substance metabolism. These processes plus the process ‘cell wall organization or bio-genesis’ showed a sign of vigorous growth and division within the transgenic plants. Except for the growth-promotion processes, ‘ion transport’ process was also identified as one of the top ten enriched bio-processes, which might provide a solid nutritional support for the striving of transgenic plants. Seven hub genes were screened within the significant phenotypic modules to illustrate the molecular mechanism further. The two up-regulated genes (*XTH* and *PR0*) are involved in the carbohydrate metabolic process and cell wall biogenesis. The five downregulated genes (*LOC107823473*, *Ntubc2*, *LOC107786522*, *LOC107761646*, *LOC107787142*) are involved in DNA repair and negative regulation of the TOR signaling. *XTH* (Xyloglucan endotransglucosylase/hydrolases) principally takes responsibility for the rearrangement of cell wall by cleavage of the β-1,4-glycosidic bonds and molecular grafting of xyloglucan chains [30]. *XTH* gene was reported to be involved in cell proliferation, cell elongation in Arabidopsis inflorescence stems, and primary root elongation [31]. The homologous gene of *XTH* was reported to promote the plant height and internode length of the transgenic tobacco plants [32]. Our finding that high expression of *XTH* was closely associated with more flowers, long roots and long stem length is consistent with the previous studies. A homologous gene of *PR0* was reported to participate in cell division [33], microsporogenesis [34] and pollen tube growth [35]. A homologous gene of *Ntubc2* was reported redundantly to repress flowering by enhancing flowering locus c (*FLC*) expression [36]. In *Arabidopsis thaliana* and related species, *FLC* is a central repressor in the flowering transition [37]. The up-regulation of *PR0* and down-regulation of *Ntubc2 *led to the flowering-promoting phenotype of transgenic lines. The homologous gene of *LOC107823473* was reported as a Reduced-Height gene [38], so *LOC107823473* down-regulated transgenic lines have longer stems than W.T. The *LOC107823473* gene also contributes to DNA repair by interacting with replication forks [39], and the homologous gene of *LOC107786522* was reported participating in DNA damage repair during somatic meiosis [40]. DNA repair is the fundamental mechanism to maintain genomic stability, and the impairment of the DNA repair system links to aging [41]. The suppression of DNA repair might be a way to save energy to facilitate rapid cell proliferation. Furthermore, it was reported that a homologue of down-regulated *LOC107786522* gene could inactivate TORC1 in amino acid starvation [42]. The TOR kinase signaling pathway has been well studied in mammals and was identified as a central regulator to promote cell proliferation and growth [43]. Recently, a similar role of TOR that integrates nutrient status and hormone signaling to regulate growth has been demonstrated in Arabidopsis [44]. Releasing the negative regulation of the TOR signaling pathway means the suppression of growth is abolished and cell proliferation is enhanced. All of the above hints suggest that the growth-promotion effect of ectopically expressed *JcTAW1* works in three aspects: (1) directly strengthening the energy metabolism and cell proliferation; (2) improving the nutrient status by enhancing ion transport processes and releasing the TOR pathway from the negative regulation, which would further promote the cell proliferation; (3) suppressing the DNA repair system to save energy and also promote the cell proliferation.

Additionally, an enhancement of drought resistance of *JcTAW1* transgenic plants was observed in our study. This may be associated with a more developed root system. Besides, the *XTH* gene was reported in response to drought and heat stress [45], so the up-regulation of *XTH* might contribute to the drought tolerance of transgenic plants.

Taken together, the current work is the first to investigate the function of the Jatropha *JcTAW1* gene in a model plant, tobacco. Our study provides a promising option for the high-yield breeding of Jatropha. The ectopically expressed *JcTAW1* can expand the  inflorescence and root architecture in tobacco. Besides, various secondary morphological traits were observed, such as higher plant height and increased drought resistance than that of W.T. From the RNA-seq analysis, *JcTAW1* seems to rearrange energy to maximize ‘cell proliferation’, but the cost might be an unstable genome or even rapid aging, which might be the reason why the expression of *TAW1* is controlled tightly in W.T. The yield-increasing effect of *TAW1* is significant. However, regarding perennial woody plants like Jatropha, a serious question is how to strike a balance between fertility and aging for overall reproduction success throughout the entire life cycle. Moreover, our study used a nonspecific CaMV 35S promoter, so *JcTAW1* expressed in the whole plant. In a further application, a flower or root-specific promoter could be chosen depending on the needs of yield or drought resistance of the target plants.

## Supplementary Information

**Additional file 1: Figure S1.** The nucleotide sequence of *JcTAW1* gene and its deduced protein sequence. The shadow represents a nuclear localization signal. **Figure S2.** Neighbor-joining based phylogenetic analysis of *JcTAW1* against all plant species. **Figure S3.** Neighbor-joining based phylogenetic analysis of OsTAW1 against its orthologs of *Jatropha curcas. ***Figure S4.** The *JcTAW1* transgenic tobacco plants exhibiting an increased flowering phenotype. **Figure S5.** The stem length of the *JcTAW1*transgenic tobacco plants were significantly higher then the WT plants. **Figure S6.** Uncropped original gel electrophoresis data. The uncropped scans of gels in Figure 1 are shown above, with a black outline to show the excerpted portion. **Table S1.** PCR primers used in this work. **Table S2.** GO enrichment analysisof enriched biological processesin interesting modules. **Table S3.** GO enrichment analysisof enriched cellular componentin interesting modules. **Table S4.** GO enrichment analysisof enriched molecular functionin interesting modules.

## Data Availability

The datasets necessary for supporting the results of this article are included in this manuscript and its additional files. The RNA sequencing reads are available in the NCBI Sequence Read Archive database (BioProject ID: PRJNA852860).
