# Supplementary material for: Ectopic expression of Jatropha curcas JcTAW1 improves the vegetative growth, yield, and drought resistance of tobacco
Source: BMC Plant Biol. 2023 Feb 4;23:77. doi: 10.1186/s12870-023-04085-2 (PMC9898971; doi:10.1186/s12870-023-04085-2)
Supplement: Supplementary file 1 — Additional file 1: Figure S1. The nucleotide sequence of JcTAW1 gene and its deduced protein sequence. The shadow represents a nuclear localization signal. Figure S2. Neighbor-joining based phylogenetic analysis of JcTAW1 against all plant species. Figure S3. Neighbor-joining based phylogenetic analysis of OsTAW1 against its orthologs of Jatropha curcas. Figure S4. The JcTAW1 transgenic tobacco plants exhibiting an increased flowering phenotype. Figure S5. The stem length of the JcTAW1transgenic tobacco plants were significantly higher then the WT plants. Figure S6. Uncropped original gel electrophoresis data. The uncropped scans of gels in Figure 1 are shown above, with a black outline to show the excerpted portion. Table S1. PCR primers used in this work. Table S2. GO enrichment analysisof enriched biological processesin interesting modules. Table S3. GO enrichment analysisof enriched cellular componentin interesting modules. Table S4. GO enrichment analysisof enriched molecular functionin interesting modules. [file 12870_2023_4085_MOESM1_ESM.pdf]

Supplementary material

Table S1 PCR primers used in this work

Primer	sequence(5'to3')	bases
Pt7Up-Fw	ACCGCGAAATTAATACGACTCAC	23
Tt7Dw-Rv	ACCGGATATAGTTCCTCCTTTCAG	24
JcTAW1-Fw	GTACATCTCTTTATCCAAACATG	23
JcTAW1-Rv	CAACACTAGAAAACATAAACCCT	23
JcTAW1-5Kn	GTTATGGTACCGACGCAACAATGGCTGCTGGCAATGGGAGTT	42
JcTAW1-3Sc	GCGGCGAGCTCACTACATGAGGAATAATC	48
JcTAW1-iFw	AgCATCgCCATCTCCTCAAC	20
JcTAW1-iRv	gCCTgTgCATCTCTCACTTC	20
Nt18S-iFw	gAAACggCTACCACATCCAAg	21
Nt18S-iRv	ggCAAATgCTTTCgCAgTTg	20
35Spro-Fw	GACGTAAGGGATGACGCACAATC	23
NosTer-Rv	GACCGGCAACAGGATTCAATC	21

```
1    ATGGCTGCTGGCAATGGGAGTTCTTCAAGAAATGATAATATATCCCCAGCATCGCCATCTCCTCAACCACCGCCAATAGCACCGCCCTCA
      M  A  A  G  N  G  S  S  S  R  N  D  N  I  S  P  A  S  P  S  P  Q  P  P  P  I  A  P  P  S
91   CTTCCACTGAGTCGTTACGAATCTCAAAAAAGACGTGACTGGAATACTTTCGGTCAATACCTAAGAAACCACCGGCCACCTTTAGCTCTG
      L  P  L  S  R  Y  E  S  Q  K  R  R  D  W  N  T  F  G  Q  Y  L  R  N  H  R  P  P  L  A  L
181  TCACGGTGCAGCGGTGCTCATGTTCTTGAATTTTTAAGATATCTTGACCAATTTGGTAAGACTAAAGTGCATAACCAAACTTGTCCATTC
      S  R  C  S  G  A  H  V  L  E  F  L  R  Y  L  D  Q  F  G  K  T  K  V  H  N  Q  T  C  P  F
271  TTTGGCCACCCTCATCCACCTGCTCCTTGCCCTTGCCCACTTAAACAAGCTTGGGGTAGCCTTGACGCGCTCATCGGACGGCTGAGAGCA
      F  G  H  P  H  P  P  A  P  C  P  C  P  L  K  Q  A  W  G  S  L  D  A  L  I  G  R  L  R  A
361  GCTTTTGAAGAGAATGGTGGCTTGCCTGAGACTAATCCGTTTGGTGCACGCGCCGTCAGACTTTATCTTAGGGAAGTGAGAGATGCACAG
      A  F  E  E  N  G  G  L  P  E  T  N  P  F  G  A  R  A  V  R  L  Y  L  R  E  V  R  D  A  Q
451  GCCAAAGCTAGAGGAATAGCTTATGAGAAGAAGAAGAGAAAGAAGCCACAACCTCAACAACAGTTACGATCTGAAAATGGTTACAATAAT
      A  K  A  R  G  I  A  Y  E  K  K  K  R  K  K  P  Q  P  Q  Q  Q  L  R  S  E  N  G  Y  N  N
541  CAAAGTCTGCAAGCTGGTGGCGCCGGTGGTGGATTTGTGGTTGAAAGAAATATGATAAACAGAGGTGTGGTTAATATGGGGAATTTGTCT
      Q  S  L  Q  A  G  G  A  G  G  G  F  V  V  E  R  N  M  I  N  R  G  V  V  N  M  G  N  L  S
631  GTATTGAACTAA
      V  L  N  *
```

Figure S1 The nucleotide sequence of *JcTAW1* gene and its deduced protein sequence. The shadow represents a nuclear localization signal

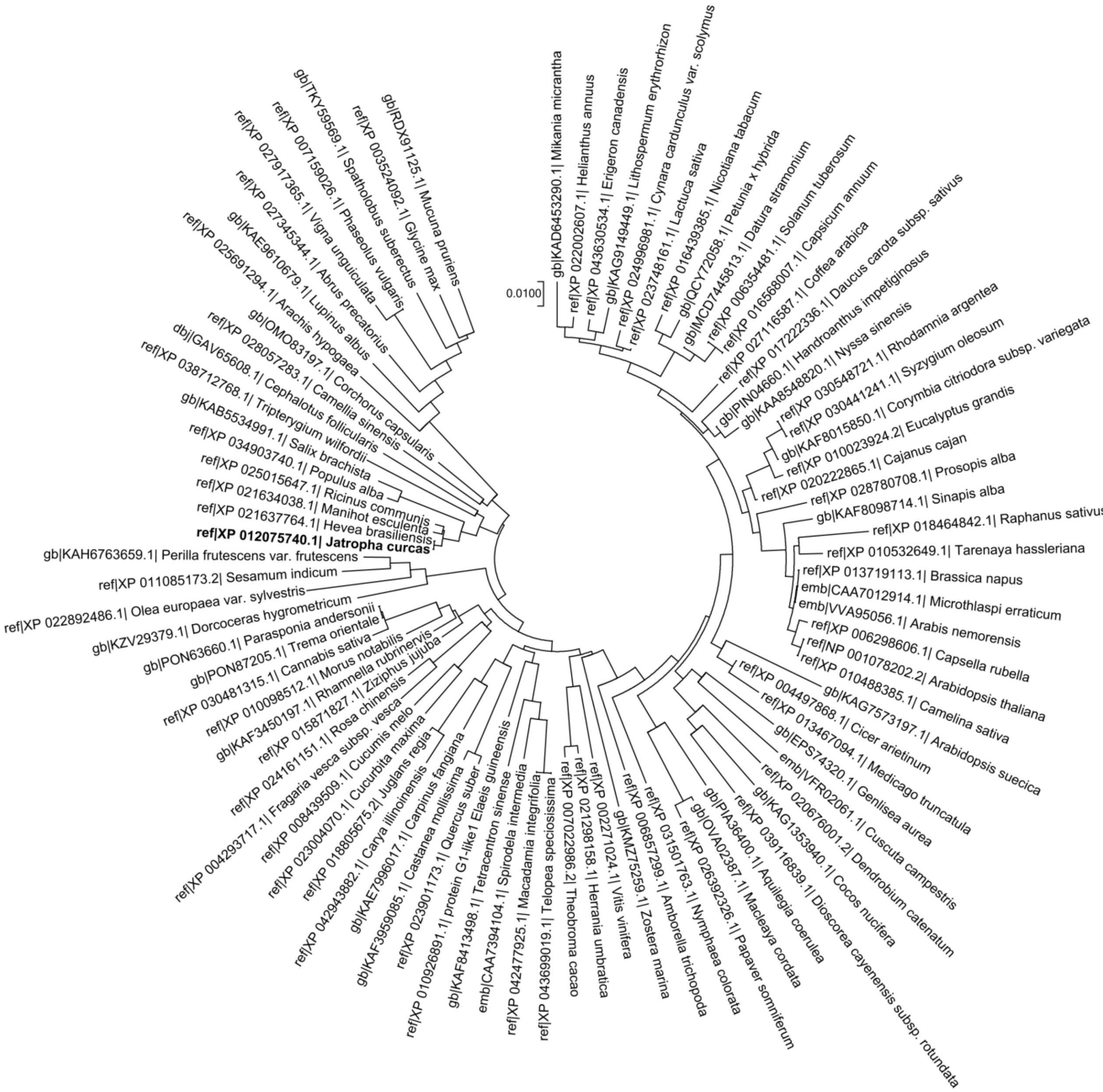

Figure S2 Neighbor-joining based phylogenetic analysis of *JcTAW1* against all plant species.

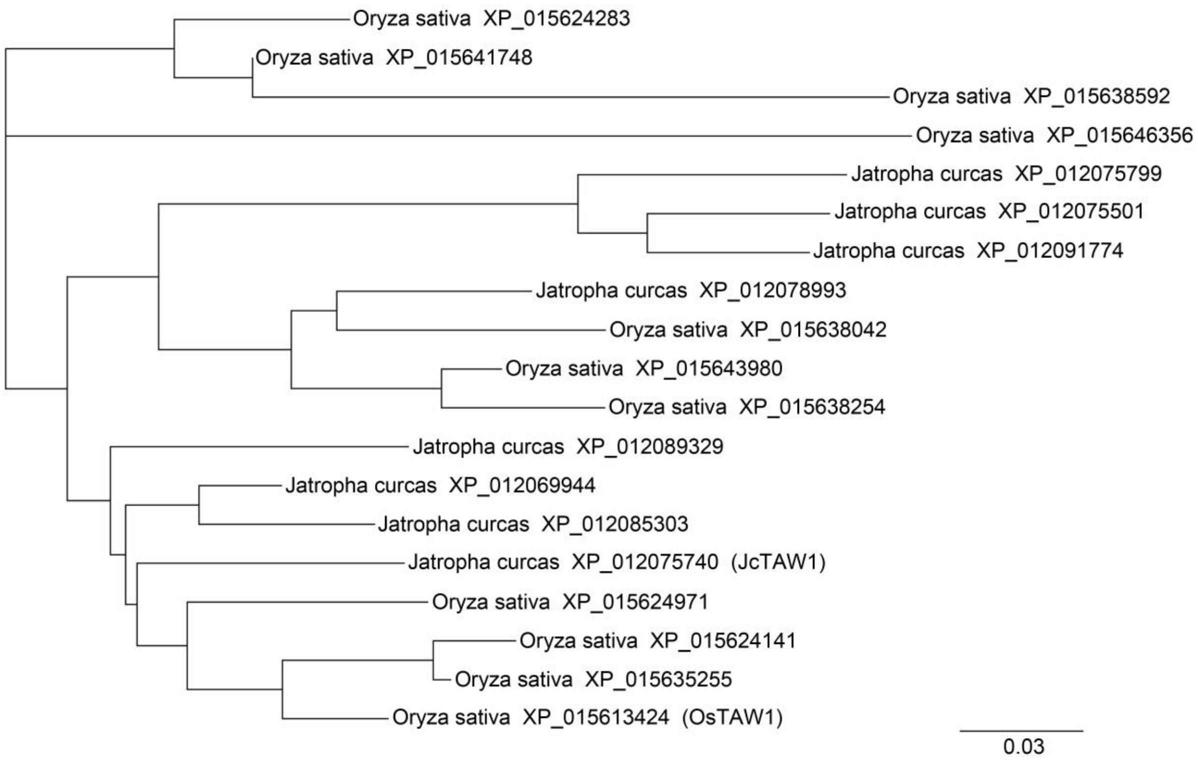

Figure S3 Neighbor-joining based phylogenetic analysis of OsTAW1 against its orthologs of *Jatropha curcas*.

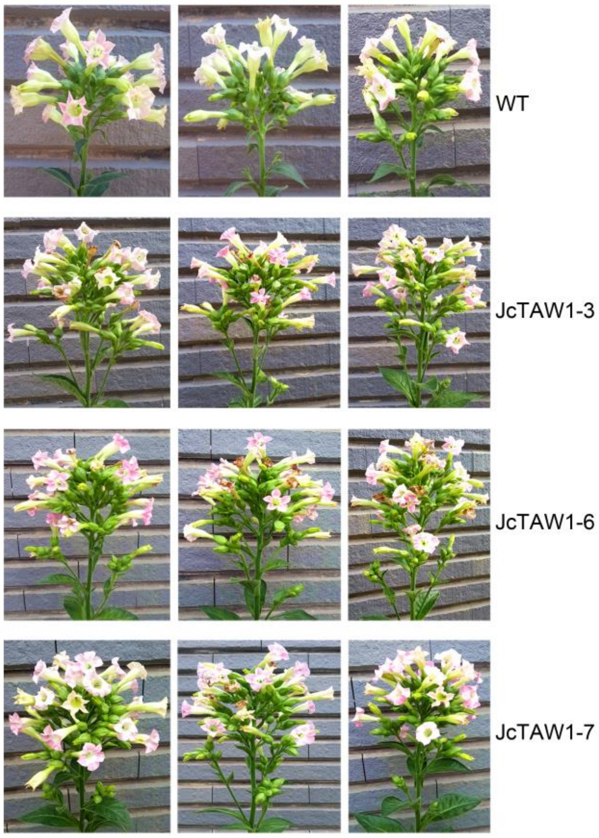

Figure S4 The *JcTAW1* transgenic tobacco plants exhibiting an increased flowering phenotype.

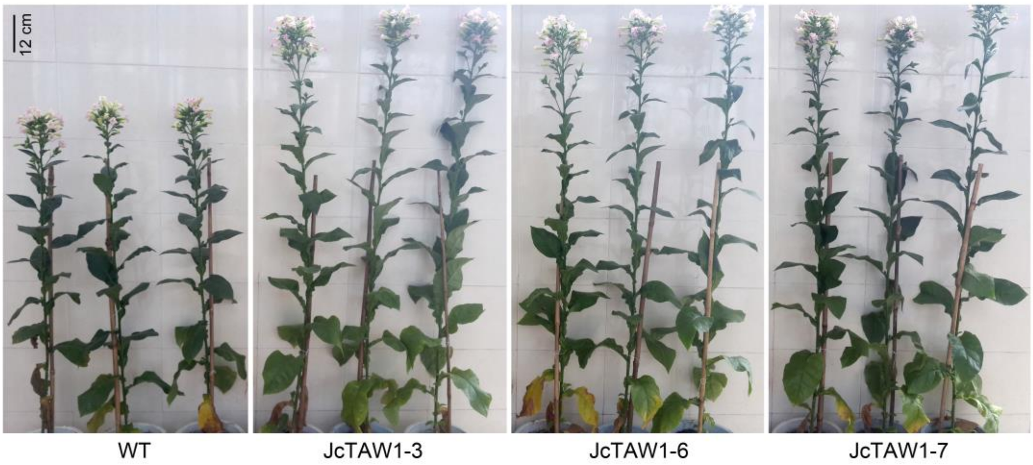

Figure S5 The stem length of the *JcTAW1* transgenic tobacco plants were significantly higher then the WT plants

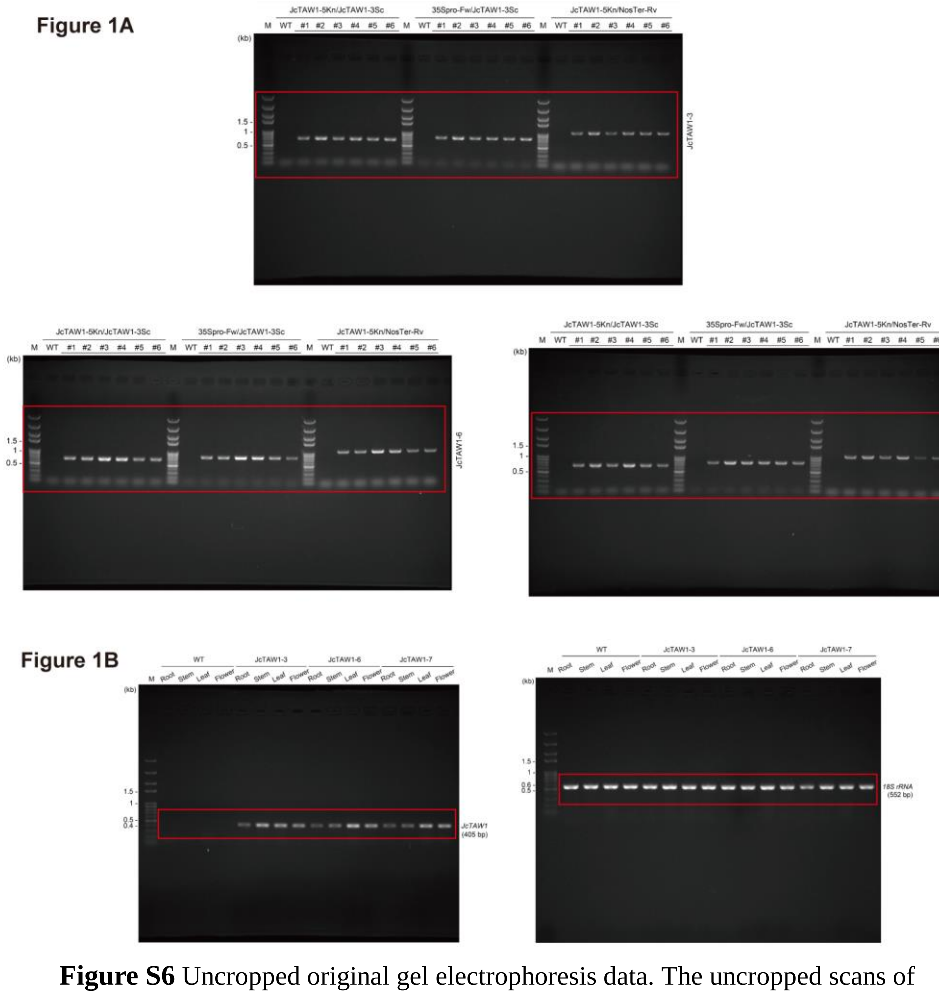

Figure S6 Uncropped original gel electrophoresis data. The uncropped scans of gels in Figure 1 are shown above, with a black outline to show the excerpted portion.

Table S2 GO enrichment analysis of enriched biological processes in interesting modules.

ID	Description	pvalue	GeneRatio
GO:0005975	carbohydrate metabolic process	8.00E-11	41/143
GO:0055114	oxidation-reduction process	6.24E-10	33/143
GO:0042592	homeostatic process	1.92E-08	32/143
GO:0006811	ion transport	2.31E-08	30/143
GO:0042221	response to chemical	3.59E-08	34/143
GO:0044262	cellular carbohydrate metabolic process	9.37E-08	25/143
GO:0006091	generation of precursor metabolites and energy	2.95E-07	26/143
GO:0016310	phosphorylation	4.35E-07	31/143
GO:0071554	cell wall organization or biogenesis	4.64E-07	17/143
GO:0007154	cell communication	1.37E-06	32/143
GO:0006979	response to oxidative stress	1.39E-06	14/143
GO:0015980	energy derivation by oxidation of organic compounds	2.15E-06	18/143
GO:0006952	defense response	2.81E-06	17/143
GO:0034599	cellular response to oxidative stress	3.14E-06	10/143
GO:0062197	cellular response to chemical stress	3.14E-06	10/143
GO:0055085	transmembrane transport	4.68E-06	24/143
GO:0071555	cell wall organization	5.45E-06	13/143
GO:0045229	external encapsulating structure organization	6.98E-06	13/143
GO:0034220	ion transmembrane transport	7.40E-06	18/143
GO:0042546	cell wall biogenesis	7.57E-06	11/143
GO:0016051	carbohydrate biosynthetic process	8.04E-06	18/143
GO:0005976	polysaccharide metabolic process	8.64E-06	16/143
GO:0006073	cellular glucan metabolic process	8.88E-06	13/143
GO:0007165	signal transduction	9.52E-06	28/143
GO:0044042	glucan metabolic process	9.99E-06	13/143
GO:0015979	photosynthesis	1.29E-05	14/143
GO:0032787	monocarboxylic acid metabolic process	1.36E-05	26/143
GO:0023052	signaling	1.44E-05	28/143
GO:0009832	plant-type cell wall biogenesis	1.45E-05	9/143
GO:0009891	positive regulation of biosynthetic process	1.69E-05	24/143
GO:0010557	positive regulation of macromolecule biosynthetic process	1.69E-05	24/143
GO:0031328	positive regulation of cellular biosynthetic process	1.69E-05	24/143
GO:0045454	cell redox homeostasis	1.74E-05	9/143
GO:0044264	cellular polysaccharide metabolic process	1.75E-05	14/143
GO:0006468	protein phosphorylation	1.82E-05	19/143
GO:0032502	developmental process	1.86E-05	28/143
GO:0044419	interspecies interaction between organisms	2.18E-05	13/143
GO:0045934	negative regulation of nucleobase-containing compound metabolic process	2.22E-05	18/143
GO:0006812	cation transport	2.29E-05	17/143
GO:0051172	negative regulation of nitrogen compound metabolic process	2.40E-05	22/143
GO:0045333	cellular respiration	2.59E-05	15/143
GO:0071669	plant-type cell wall organization or biogenesis	3.42E-05	9/143

GO:0000278	mitotic cell cycle	3.44E-05	25/143
GO:0034637	cellular carbohydrate biosynthetic process	3.65E-05	13/143
GO:0015977	carbon fixation	3.77E-05	6/143
GO:1901615	organic hydroxy compound metabolic process	3.85E-05	22/143
GO:0010558	negative regulation of macromolecule biosynthetic process	3.88E-05	19/143
GO:2000113	negative regulation of cellular macromolecule biosynthetic process	3.88E-05	19/143
GO:1901617	organic hydroxy compound biosynthetic process	3.91E-05	17/143
GO:0007049	cell cycle	3.93E-05	32/143

Table S3 GO enrichment analysis of enriched cellular component in interesting modules.

ID	Description	pvalue	GeneRatio
GO:0005886	plasma membrane	1.24E-14	51/143
GO:0005576	extracellular region	3.53E-11	28/143
GO:0031984	organelle subcompartment	3.62E-11	34/143
GO:0031967	organelle envelope	1.02E-07	37/143
GO:0031975	envelope	1.02E-07	37/143
GO:0009534	chloroplast thylakoid	1.31E-07	22/143
GO:0031976	plastid thylakoid	1.31E-07	22/143
GO:0009579	thylakoid	1.42E-07	23/143
GO:0005794	Golgi apparatus	1.54E-07	30/143
GO:0048046	apoplast	1.66E-06	10/143
GO:0009535	chloroplast thylakoid membrane	2.63E-06	18/143
GO:0055035	plastid thylakoid membrane	2.63E-06	18/143
GO:0042651	thylakoid membrane	3.19E-06	18/143
GO:0034357	photosynthetic membrane	3.87E-06	18/143
GO:0005783	endoplasmic reticulum	7.33E-06	27/143
GO:0005615	extracellular space	8.35E-06	10/143
GO:0005740	mitochondrial envelope	8.82E-06	23/143
GO:0009526	plastid envelope	1.35E-05	18/143
GO:0019866	organelle inner membrane	1.56E-05	20/143
GO:0031966	mitochondrial membrane	1.59E-05	22/143
GO:0005743	mitochondrial inner membrane	1.80E-05	19/143
GO:0098791	Golgi apparatus subcompartment	2.02E-05	14/143
GO:0005802	trans-Golgi network	2.52E-05	12/143
GO:0098796	membrane protein complex	3.58E-05	31/143
GO:0009941	chloroplast envelope	5.66E-05	16/143
GO:0005789	endoplasmic reticulum membrane	0.000105354	25/143
GO:0042175	nuclear outer membrane-endoplasmic reticulum membrane network	0.000105354	25/143
GO:0005773	vacuole	0.000147194	20/143
GO:0031301	integral component of organelle membrane	0.000151097	19/143
GO:0031300	intrinsic component of organelle membrane	0.000170584	19/143
GO:0042170	plastid membrane	0.000232258	12/143
GO:0031226	intrinsic component of plasma membrane	0.000314837	11/143
GO:0005938	cell cortex	0.000344184	9/143

GO:0090575	RNA polymerase II transcription regulator complex	0.000619586	10/143
GO:0005759	mitochondrial matrix	0.000636657	14/143
GO:0019867	outer membrane	0.000671743	9/143
GO:0031968	organelle outer membrane	0.000671743	9/143
GO:0030880	RNA polymerase complex	0.000680568	14/143
GO:0009521	photosystem	0.000782837	7/143
GO:0031969	chloroplast membrane	0.001065272	10/143
GO:0005618	cell wall	0.001068465	8/143
GO:0030312	external encapsulating structure	0.001068465	8/143
GO:0009506	plasmodesma	0.001325373	8/143
GO:0055044	symplast	0.001325373	8/143
GO:0016591	RNA polymerase II, holoenzyme	0.001327747	11/143
GO:0061695	transferase complex, transferring phosphorus-containing groups	0.001462067	19/143
GO:0070469	respirasome	0.001629242	8/143
GO:1990204	oxidoreductase complex	0.001656129	11/143
GO:0031225	anchored component of membrane	0.001707864	5/143
GO:0005654	nucleoplasm	0.001838463	23/143

Table S4 GO enrichment analysis of enriched molecular function in interesting modules.

ID	Description	pvalue	GeneRatio
GO:0098772	molecular function regulator	3.72E-08	40/148
GO:0015318	inorganic molecular entity transmembrane transporter activity	6.54E-08	29/148
GO:0015075	ion transmembrane transporter activity	1.90E-07	29/148
GO:0008324	cation transmembrane transporter activity	5.74E-07	23/148
GO:0004497	monooxygenase activity	8.46E-07	14/148
GO:0030234	enzyme regulator activity	1.07E-06	27/148
GO:0016209	antioxidant activity	1.07E-06	12/148
GO:0016651	oxidoreductase activity, acting on NAD(P)H	1.36E-06	13/148
GO:0005215	transporter activity	1.98E-06	34/148
GO:0022857	transmembrane transporter activity	2.12E-06	32/148
GO:0050660	flavin adenine dinucleotide binding	5.67E-06	15/148
GO:0046527	glucosyltransferase activity	6.38E-06	11/148
GO:0004362	glutathione-disulfide reductase activity	1.00E-05	5/148
GO:0009055	electron transfer activity	1.00E-05	14/148
GO:0015078	proton transmembrane transporter activity	1.08E-05	15/148
GO:0022890	inorganic cation transmembrane transporter activity	1.19E-05	20/148
GO:0019207	kinase regulator activity	1.46E-05	12/148
GO:0005509	calcium ion binding	1.47E-05	16/148
GO:0015077	monovalent inorganic cation transmembrane transporter activity	1.77E-05	16/148
GO:0008309	double-stranded DNA exodeoxyribonuclease activity	2.98E-05	5/148
GO:0008311	double-stranded DNA 3'-5' exodeoxyribonuclease activity	2.98E-05	5/148
GO:0015037	peptide disulfide oxidoreductase activity	4.71E-05	5/148
GO:0015038	glutathione disulfide oxidoreductase activity	4.71E-05	5/148
GO:0016709	oxidoreductase activity, acting on paired donors, with incorporation or	5.64E-05	9/148

	reduction of molecular oxygen, NAD(P)H as one donor, and incorporation of one atom of oxygen		
GO:0004674	protein serine/threonine kinase activity	6.36E-05	16/148
GO:0046983	protein dimerization activity	6.47E-05	15/148
GO:0000981	DNA-binding transcription factor activity, RNA polymerase II-specific	8.25E-05	7/148
GO:0004672	protein kinase activity	9.16E-05	18/148
GO:0016798	hydrolase activity, acting on glycosyl bonds	0.000124544	17/148
GO:0022804	active transmembrane transporter activity	0.000136336	18/148
GO:0016829	lyase activity	0.000161072	26/148
GO:0005506	iron ion binding	0.000190843	14/148
GO:0035251	UDP-glucosyltransferase activity	0.000261305	8/148
GO:0004528	phosphodiesterase I activity	0.00027264	5/148
GO:0016758	transferase activity, transferring hexosyl groups	0.000290338	16/148
GO:0051536	iron-sulfur cluster binding	0.000321934	18/148
GO:0051540	metal cluster binding	0.000321934	18/148
GO:0046906	tetrapyrrole binding	0.000325616	14/148
GO:0003700	DNA-binding transcription factor activity	0.000367197	11/148
GO:0015036	disulfide oxidoreductase activity	0.000424432	7/148
GO:0004519	endonuclease activity	0.00044514	17/148
GO:0008081	phosphoric diester hydrolase activity	0.000450532	8/148
GO:0016668	oxidoreductase activity, acting on a sulfur group of donors, NAD(P) as acceptor	0.000466512	5/148
GO:0022853	active ion transmembrane transporter activity	0.00052553	12/148
GO:0004611	phosphoenolpyruvate carboxykinase activity	0.000543393	4/148
GO:0016984	ribulose-bisphosphate carboxylase activity	0.000543393	4/148
GO:0016538	cyclin-dependent protein serine/threonine kinase regulator activity	0.000550314	6/148
GO:0019887	protein kinase regulator activity	0.000586295	9/148
GO:0016830	carbon-carbon lyase activity	0.000612812	14/148
GO:0004386	helicase activity	0.000662063	15/148
